# A study on the value of computer-assisted assessment for SPECT/CT-scans in sentinel lymph node diagnostics of penile cancer as well as clinical reliability and morbidity of this procedure

**DOI:** 10.1186/s40644-016-0087-z

**Published:** 2016-09-07

**Authors:** Ulf Lützen, Carsten Maik Naumann, Marlies Marx, Yi Zhao, Michael Jüptner, René Baumann, László Papp, Norbert Zsótér, Alexey Aksenov, Klaus-Peter Jünemann, Maaz Zuhayra

**Affiliations:** 1 Department of Nuclear medicine, Molecular Imaging Diagnostics and Therapy, University Hospital Schleswig Holstein, Campus Kiel, Feldstr. 21 (Haus 50), 24105 Kiel, Germany; 2 Department of Urology and Pediatric Urology, University Hospital Schleswig Holstein, Campus Kiel, Kiel, Germany; 3 Department of Radiotherapy, University Hospital Schleswig Holstein, Campus Kiel, Kiel, Germany; 4 Division of Nuclear Medicine, Medical University of Vienna, Vienna, Austria; 5 Mediso Ltd., Budapest, Hungary

**Keywords:** SPECT/CT, Computer-assisted assessment, Penile cancer, Sentinel lymph nodes, Lymph node biopsy, Tc 99 m-nanocolloid, CAD

## Abstract

**Background:**

Because of the increasing importance of computer-assisted post processing of image data in modern medical diagnostic we studied the value of an algorithm for assessment of single photon emission computed tomography/computed tomography (SPECT/CT)-data, which has been used for the first time for lymph node staging in penile cancer with non-palpable inguinal lymph nodes. In the guidelines of the relevant international expert societies, sentinel lymph node-biopsy (SLNB) is recommended as a diagnostic method of choice. The aim of this study is to evaluate the value of the afore-mentioned algorithm and in the clinical context the reliability and the associated morbidity of this procedure.

**Methods:**

Between 2008 and 2015, 25 patients with invasive penile cancer and inconspicuous inguinal lymph node status underwent SLNB after application of the radiotracer Tc-99m labelled nanocolloid. We recorded in a prospective approach the reliability and the complication rate of the procedure. In addition, we evaluated the results of an algorithm for SPECT/CT-data assessment of these patients.

**Results:**

SLNB was carried out in 44 groins of 25 patients. In three patients, inguinal lymph node metastases were detected via SLNB. In one patient, bilateral lymph node recurrence of the groins occurred after negative SLNB. There was a false-negative rate of 4 % in relation to the number of patients (1/25), resp. 4.5 % in relation to the number of groins (2/44). Morbidity was 4 % in relation to the number of patients (1/25), resp. 2.3 % in relation to the number of groins (1/44). The results of computer-assisted assessment of SPECT/CT data for sentinel lymph node (SLN)-diagnostics demonstrated high sensitivity of 88.8 % and specificity of 86.7 %.

**Conclusions:**

SLNB is a very reliable method, associated with low morbidity. Computer-assisted assessment of SPECT/CT data of the SLN-diagnostics shows high sensitivity and specificity. While it cannot replace the assessment by medical experts, it can still provide substantial supplement and assistance.

## Background

Application of Tc-99m-labelled lymphogenic tracers for pre- and intraoperative detection of sentinel lymph node (SLN) is an internationally established standard procedure, not only in malignant melanoma and breast cancer, but also in penile cancer with an intermediate or higher degree of differentiation and a non-palpable inguinal lymph node status, and it is recommended in the guidelines of international expert societies [1–5]. During the development phase of this staging method, the Dutch workgroup around Horenblas, but also other workgroups, reported false negative rates of ca. 15 % [6, 7]. Data from a meta-analysis by Neto et al. require a critical view [8]. These include studies examining the results of sentinel lymph node-biopsy (SLNB) in patients with palpable inguinal lymph nodes. SLNB is not recommended by the national and international expert society guidelines in patients with palpable inguinal lymph nodes due to its high unreliability [3].

But only in a few countries such as the United Kingdom and the Netherlands SLNB in penile cancer is established as a standard procedure. In other countries including Germany this procedure is used neither widely nor regularly.

The reason for the lack of wide application in this entity might also be found in the high methodical demands of this method, which requires an experienced interdisciplinary team, consisting of experts in nuclear medicine, urologists and pathologists including the necessary expertise and equipment. Apart from the gamma probe that urologists employ intraoperatively, hybrid devices enabling both functional and morphological imaging is used, primarily preoperatively, for modern lymph-scintigraphical diagnostics.

The advantages of this combined imaging device over conventional planar scintigraphy (used in the procedure to date) lies in the improvement of diagnostics through co-registration of the anatomical and functional images, and thus better detection and more exact localization of the findings in SLN diagnostics due to augmented image information [9–15]. Fusion of both imaging methods helps to improve anatomical allocation of the radio-labelled lymph nodes enables improved preoperative localization and operative planning. Furthermore, preoperative SLN imaging with single photon emission computed tomography/computed tomography (SPECT/CT) is also beneficial from a surgical point of view in various tumor entities: on the one hand, selective SLNB has helped to decrease postoperative morbidity after oncological interventions; moreover, it offers clinically relevant information regarding optimal surgical access and the vicinity of important anatomical structures like vessels and nerves [7, 21]. On the other hand, studies have also shown the intraoperative advantages of exact preoperative imaging via SPECT/CT in SLNB. Both for open surgery and laparoscopic SLNB, studies have demonstrated significantly higher numbers of intraoperatively identified lymph nodes [22, 23]. Compared to intraoperative SLN detection by a gamma probe alone, preoperative hybrid imaging has helped to shorten the operation time of SLNB significantly [24]. In consideration of the results collected in the afore-mentioned, studies it becomes clear that precise and complete preoperative SLN diagnostics with SPECT/CT is of high clinical relevance for pre- and intraoperative SLN detection, in many tumor entities.

Hybrid imaging, not only in preoperative SLN-imaging, generates much larger amounts of image data. Therefore, a downside of hybrid imaging has to be seen in the large amounts of generated image data, the interpretation and assessment of which can be very time-consuming and requires expertise both in nuclear medicine and in radiology. Thanks to modern and potent information technology (IT) systems, it is close at hand to use specialized systems for helping the experts analyze and assess these large amounts of digital images. In image-related medical disciplines, this has prompted the evolution of special image processing software, developed to help the medical expert assess large amounts of digital image data in less time to achieve better diagnostic results. The literature reports studies on software applications and analyses of lung scintigraphy for embolism diagnostics [16], and for automated assessment of fluor-deoxyglucose positron-emission-computed-tomography/computertomography (FDG-PET/CT)-examinations in non-small-cell bronchial carcinoma [17]. Moreover, programs for post processing in nuclear medical imaging are now commercially available. For example, programs for analysis of skeletal and myocardial perfusion scintigraphy from EXINI Dignostics (Lund, Sweden); or computer-assisted processing of pre synaptic dopamin uptake transporter-scintigraphy of the brain can be performed via “Statistical Parametric Mapping” (SPM)-programs in patients with Parkinson syndrome.

But to our knowledge, data for the application of post-processing programs for SLN-diagnostics in penile cancer are not available in the literature yet. The aim of this prospective study is for the first time to evaluate a program for assessment of preoperative SPECT/CT-imaging to detect radio-labelled sentinel lymph nodes in this tumor entity, and to analyze the reliability and morbidity of SLNB with a view to assessing if the use of the described post-processing program may influence the reliability and morbidity of this procedure in our cohort.

## Methods

Between 2008 and 2015, 25 patients with histologically proven, invasive penile cancer and inconspicuous inguinal lymph node status underwent SLNB after application of the radiotracer Tc-99m-labelled nanocolloid. For all enrolled patients, we performed preoperative SPECT/CT-scans. The applied tracer is a pure gamma emitter with a half-life of 6 h and energy of 141 keV.

All enrolled patients additionally underwent a physical examination including palpatory assessment of the lymph node status and ultrasound screening of the inguinal region prior to surgery. Section images via magnetic resonance imaging (MRI) and computer tomography (CT) of the pelvic region including the groins were performed as a facultative measure.

**Fig. 1:**
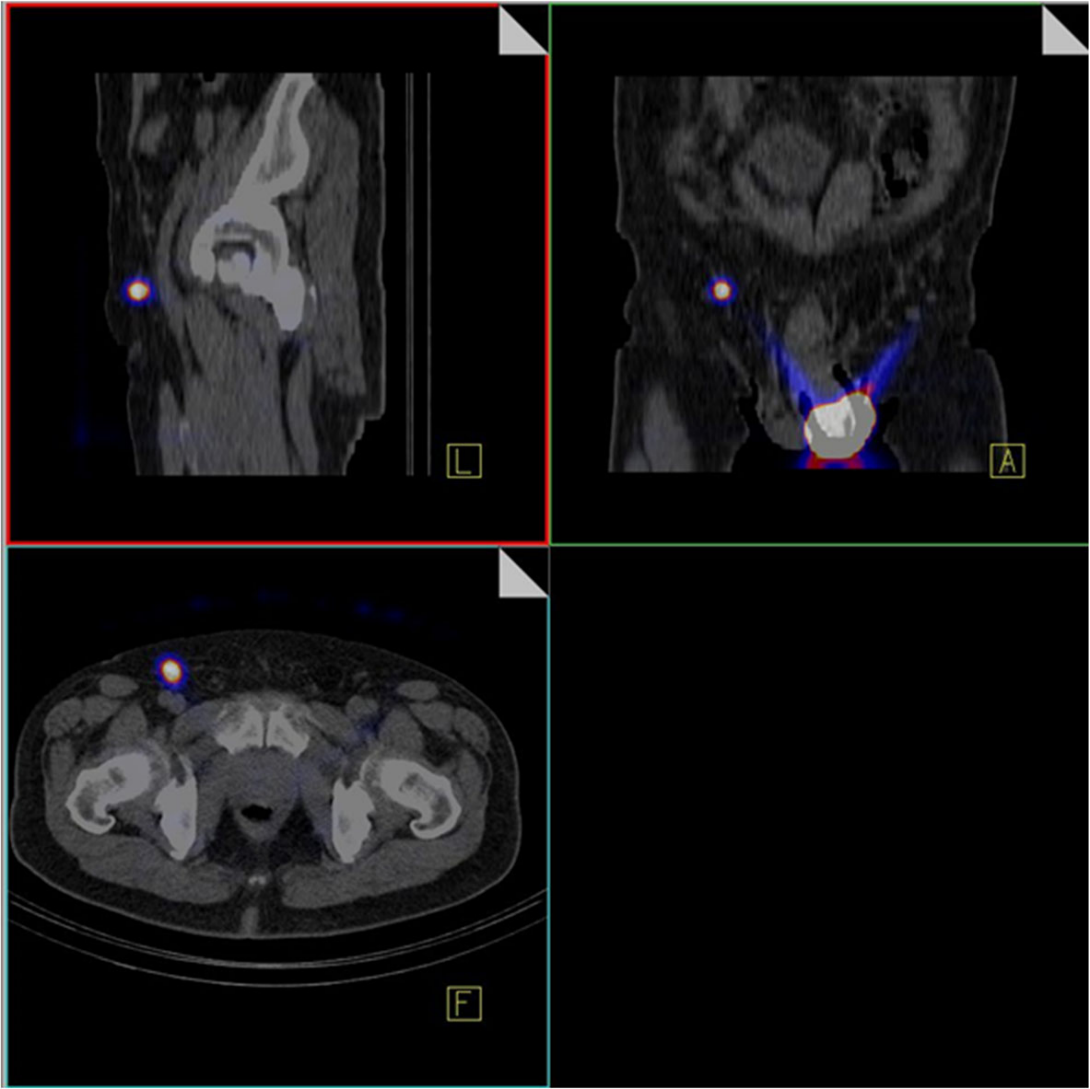
Preoperative visualization of sentinel lymph nodes via SPECT/CT: Evidence of so-called “hot spots” in the right inguinal region as well as in the periphery of the tumor (application site)

Sentinel lymph nodes (SLN) are by definition the first lymph nodes draining the primary tumor and are thus the most likely to receive metastatic spreading first. Lymphatic mapping allows surgeons to locate and excise the sentinel node by preoperative scintigraphy imaging and/or intraoperative identification using a gamma probe. However, these SLN detection methods cannot predict if the node harbours metastatic cells [18]. In our study, we have considered the lymph node with the highest tracer uptake to be the SLN of the first order, which must be located in the typical region for metastatic spread of this tumor entity (i. e. in the groins). All other radio-labeled lymph nodes are SLN of the second or higher orders, or so called echelon nodes. The software identifies all “hot nodes” and offers a list of potentially true and false findings (hot spots) in the order of calculated probabilities. The probability value is derived from both SPECT and CT indices to determine how likely “true” or “false” the given hot spot is. Intraoperatively, the radioactive inguinal lymph nodes were identified by using a handheld gamma probe. No pelvic lymph nodes were removed because only the groins are typical SLN regions. Nuclear medical visualization of the SLN was done following the so-called two-day protocol [19, 20]. On the preoperative day, the patients underwent intradermal, peritumoral injection of the radio tracer in local anesthesia. We applied an overall activity of 150 MBq Tc-99m-labelled nanocolloid. We performed lymphatic drainage scintigraphies: either on the day of injecting the radio nuclide at least one hour p. i., − or, in case of lacking or retarded lymphatic drainage, on the morning before the intervention. We acquired SPECT/CT scans of the lower abdomen and pelvis including the inguinal region by means of a twin head SPECT/CT-hybrid camera (Siemens, Symbia T and Symbia Intevo). Acquisition of SPECT scans was done using Low Energy High Resolution (LEHR) -collimators in a 128 x 128 matrix, 90 views at 12 s acquisition intervals, in a continuous mode and at a 180° detector configuration. Reconstruction of SPECT data was done via Flash 3D-iterative reconstruction with eight iterations and 16 subsets and a Gaussian filter of eight mm. CT data were acquired in a so-called low-dose technique: tube voltage 130 kV, pitch factor 1.5, reference-mAs 17, layer thickness 5 mm, collimation 2 x 4 mm. The functional imaging scans were fused with co-registered CT scans (Fig. 1). Morphological imaging enabled attenuation correction of the emission data as well as easier anatomical allocation of the radio-marked lymph nodes. All images were saved in the DICOM format.

Subsequent to imaging process, the SLN were identified and marked with a Co-57 pen as well as a felt-tip pen on the skin surface. In addition, patent-blue was injected directly preoperatively intradermally resp. peritumorally. Intraoperative identification of the SLN was thus possible both visually via the patent blue staining, as well as by measuring radioactivity with the gamma probe. Complete histopathological preparation of the SLNs was done in 100 micrometer (μm) sections with additional hematoxylin-eosin staining.

### Computer-assisted assessment of SPECT/CT data

For evaluation of the computer-assisted assessment the underlying SPECT/CT data sets of lymphatic drainage scintigraphy of the enrolled patients were initially analyzed and consensually assessed by an expert in nuclear medicine and a combined expert in radiology and nuclear medicine. Subsequently, the data sets were assessed exclusively by the Software (InterView FUSION/Mediso) to provide classification of each detected SPECT hot spot individually. Finally, the computer-assessed findings were corrected by the afore-mentioned experts. In each of the mentioned analyses, the findings were classified according to the respective side (left/right) and their anatomical position as either inguinal or secondary (parailiac and paraaortal) lymphatic drainage regions. The results of the respective assessments were analyzed statistically; sensitivity, specificity, the false-positive rate and the false-negative rate were calculated in a crosstab with the respective algorithms. For software-based assessment and experts (re-)assessment, we used the early recordings of the images (1 h p. i.) if they showed radioactive lymph nodes. Otherwise, the later acquired images, (17 h p. i.), were used for both the computer-based analysis and the expert assessment, regardless of whether radioactive lymph nodes could be visualized in these images. Finally, we measured the time required for the respective assessment modes, i. e. the experts assessment alone and the software assessment plus the required expert correction time.

The workflow of this computer-assisted assessment program consists chiefly of the following three steps:

1. Segmentation of SPECT data,
2. Segmentation of CT data,
3. Classification of results (hot nodes/lymph nodes)

**Fig. 2:**
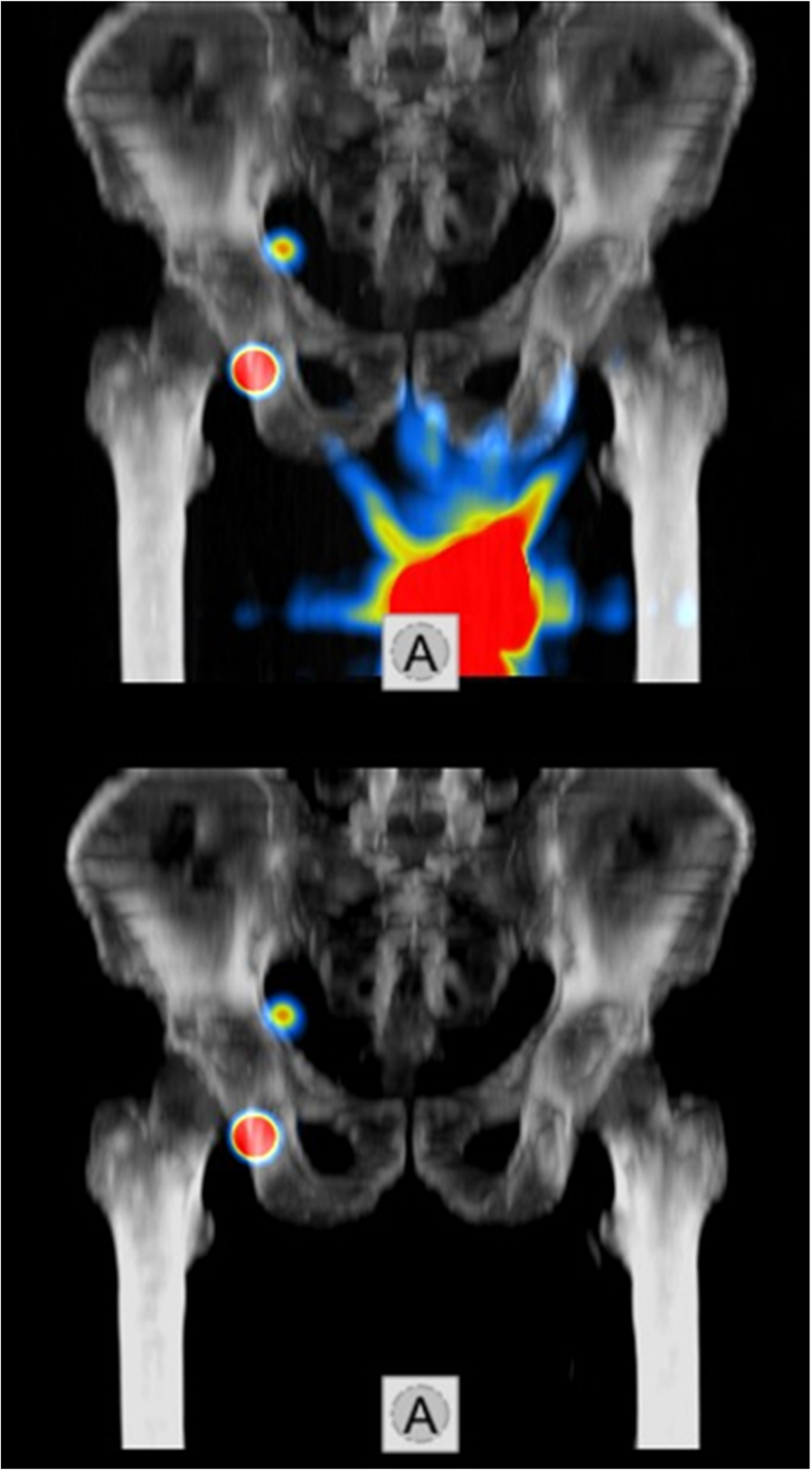
Preoperative 3D-MIP-Fusion imaging of pelvic SPECT/CT in a patient prior to application of the program (*above*) and after application of the method with elimination of the tumor region/application site (*below*) as well unchanged visualization of the SLN (hot spots) in the right inguinal region

Due to the initially different spatial resolutions of SPECT (4 × 4 × 4 mm) and CT (1 × 1 × 5 mm) the respective image lattice geometry were resampled into a uniform voxel format of 1 × 1 × 1 mm to enable voxel wise computer-assisted analysis. To this end, a cubic thin plate (b-spline) interpolation was used both for SPECT and for CT leading to increase of the smoothing effect [21]. Nevertheless this known side-effect was an acceptable trade-off in return to be able to perform voxel wise analysis. For improvement of the interpretability, the background noise of functional imaging was removed from SPECT data by the means of appropriate semi-quantitative threshold values (all values < 1 % of the injected activity). Figure 2 shows a fused SPECT/CT maximum-intensity-projection (MIP)-3D-image after application of the tracer.

### SPECT Segmentation

**Fig. 3:**
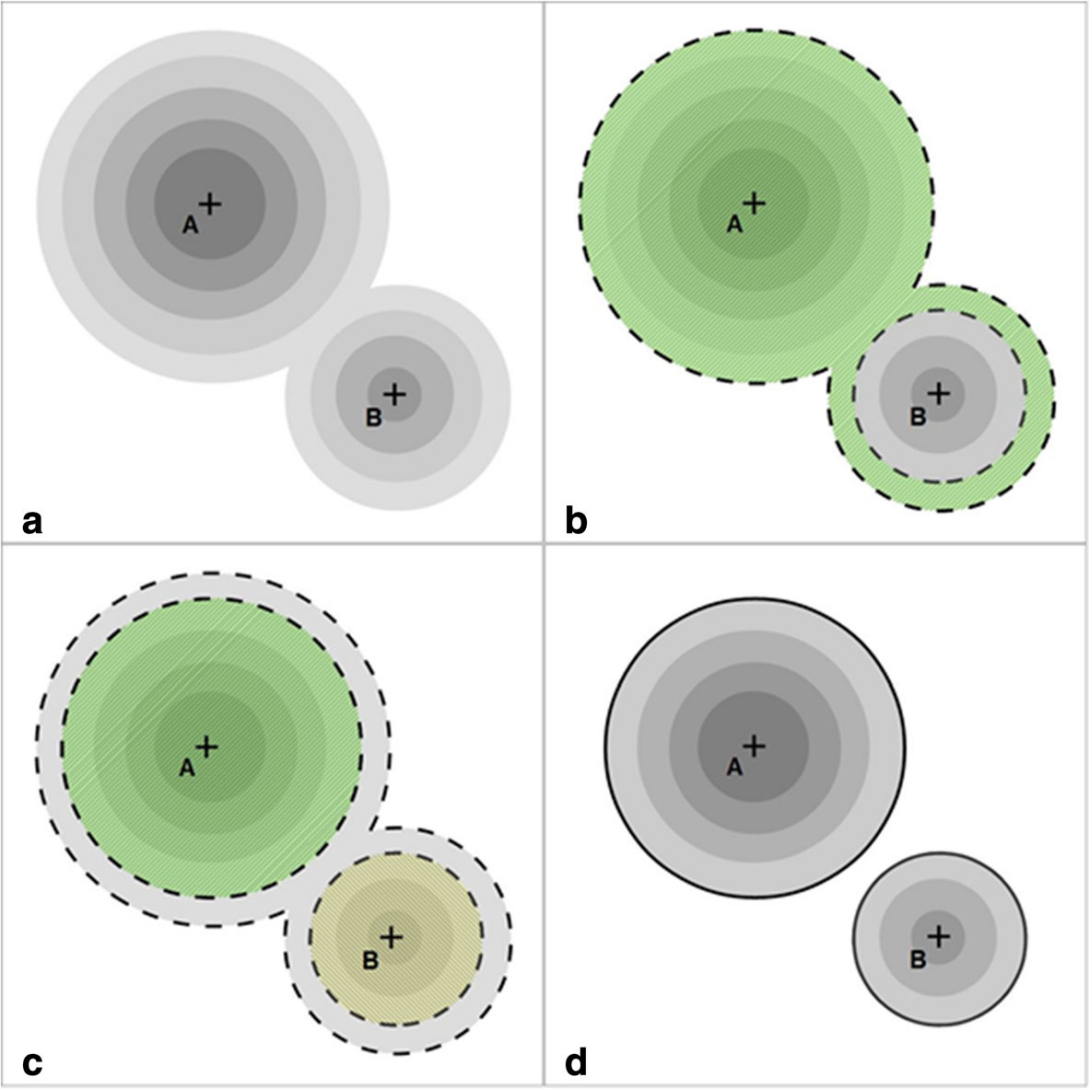
Schematic representation of hot spot detection and segmentation by SPECT in a 2D example. **a** Original image with two hot spots with their respective local maxima (“A” and “B”). **b** Result of region growing from local maxima “A” (borders with *dashed contour*). Note that the bottom of the smaller region is also absorbed as it met the region growing criteria. **c** Result of the region growing from local maxima “B” (borders with *dashed contour*). Note that comon voxels were excluded from both regions. **d** Segmented regions (*black contour*)

For definition for the findings (hot spots) in the SPECT a moving 3 × 3 × 3 voxel mask was applied in order to detect local maxima positions serving as seed point for segmentation. A recursive region-growing algorithm was used in all detected focus areas (hot spots). Employing this region-growing process, every defined focus area was compared with its 26 neighboring voxels in an iterative process to achieve exact spatial allocation of the findings and thus identification of the focus areas (hot spots). If, in this process, a neighboring voxel was smaller or equal to the actual voxel value, it was allocated to the latter. If, however, there was an increase of the value of the neighboring voxels, it was not included in the give region. If a voxel met the region growing criteria but it was already the member of another already segmented region, it was signed as common voxel. After all hot spots were individually segmented these common regions were deleted from the regions. In this way we were able to ensure that no hot spot was lost or absorbed by the neighboring voxels and that none of them touched one another on the voxel level. Figure 3 shows a graphical representation of the SPECT segmentation steps. As the segmentation approach searched for local maxima in the smallest possible kernel in resampled images, it was ensured that neighboring local maxima voxels with at least one lower voxel value in between could be identified as separate regions. Hence, the only limitation of this approach was the spatial resolution of the SPECT imaging technology. If neighboring hot spots could not be distinguished in the original SPECT image, i. e. if it showed no multiple, but only one local maximum position, the program treated them as a single larger hot spot.

### CT Segmentation

**Fig. 4:**
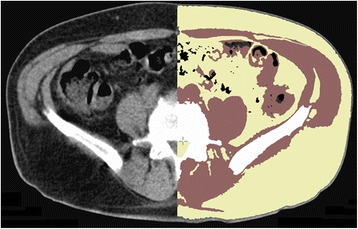
Axial CT image of the abdomen: *Left side* of the image: original scan/*Right side* of the image: segmented CT image with staining of the different tissue types (air = *black*, fat = *yellow*, muscle = *red*, and bone = *white*)

In consideration of anatomical and physiological characteristics, it is possible to define certain criteria for the localization of lymph nodes, which qualify the findings as either potentially true or definitely false. Lymph nodes cannot be located extracorporally, in parenchymous organs or in bones. SLNs consist of lymphatic tissue and are embedded in fatty and connective tissue. Therefore, the CT data were segmented according to their respective tissue type, i. e. according to their density value as Hounsfield units (HU) and classified as four different types: air, fatty tissue, muscle and bone. For further statistical analysis, we allocated the numbers 0–3 resp. colors to these tissue types. As “bone” has no homogeneous morphological structure, the bone region was mathematically replaced by post-processed one generated by morphological closing operation [22]. Table 1 shows the resulting criteria for segmentation of tissue types according to HU. Figure 4 shows an axial CT image of the abdomen, on the left-hand side as a representation the original image and on the right-hand side of the segmented image. The reason for this approach was that our CT images were low-dose with a 5 mm transaxial resolution. Due to this, lymphatic tissue inside true lymph node spots could not be visualized by the applied CT imaging technology. Thus, the CT voxel HU values inside the hot spots were no useful features for the classification. Nevertheless, we found that the direct vicinity of the hot spots was a stable feature to determine the type of the surrounding tissue, thus assisting the final classification of the hot spots. Only those CT-data that were relevant for the afore-mentioned segmentation and identification of hot spots in the emission data were considered by the algorithm. However, further information such as morphological aspects or findings was not considered by the program in any way.

**Table 1:** Segmentation of tissue types after HE

Classification/color	HE min.	HE max.	Tissue type
0/black	< (min.)	- 200	Air
1/yellow	- 200	- 20	Fat
2/red	- 20	150	Muscle
3/white	150	> (max.)	Bone

### Classification of findings (hot spots/lymph nodes)

**Fig. 5:**
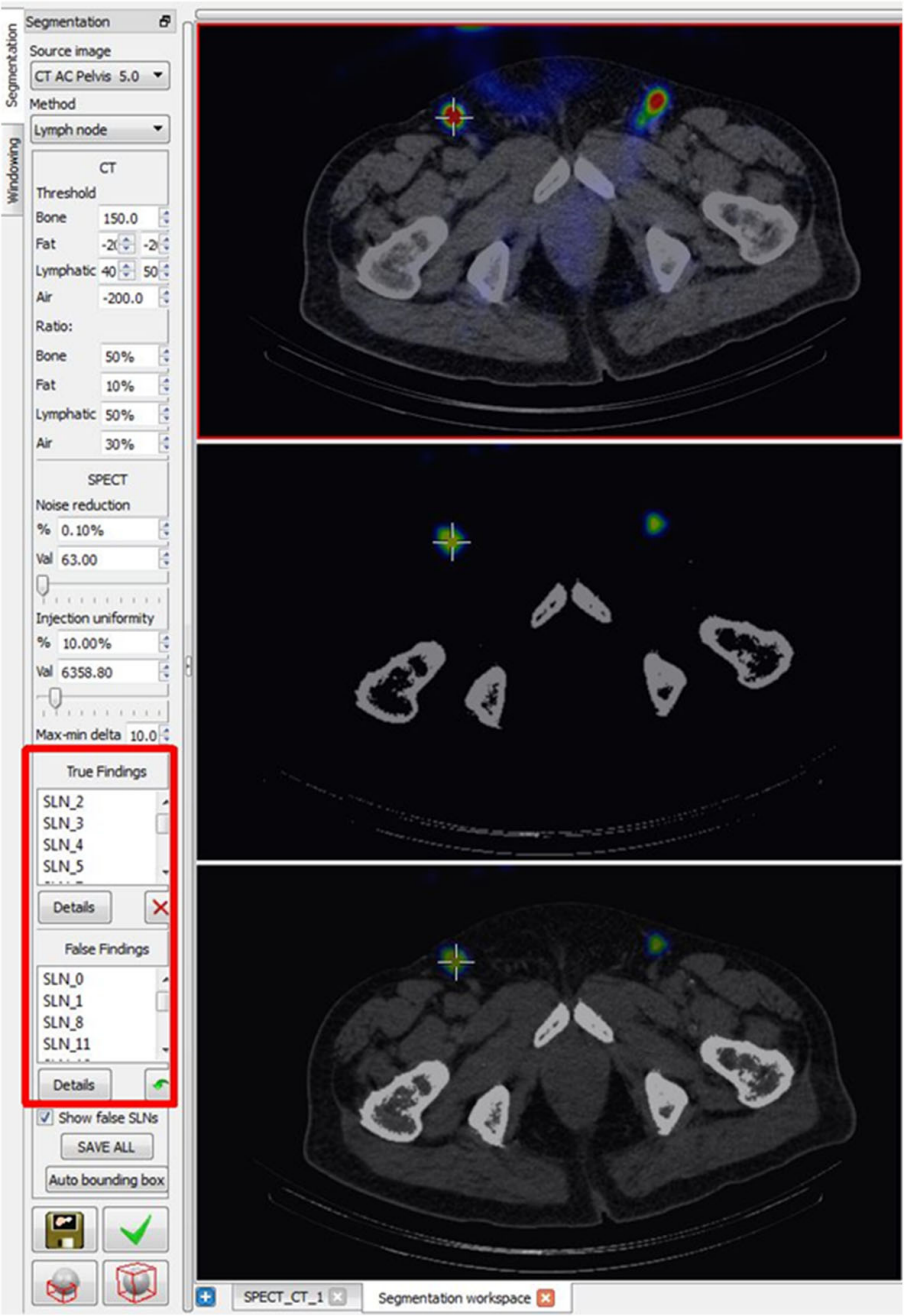
Excerpt from the screen display of the software (InterView FUSION/Mediso) with axial CT scan of the abdomen. The hot spot marked with a cross on the *right* inguinal region was identified as a “true” finding by the software and thus as a true SLN. On the *lower left* periphery of the image you can find the lists of the “true” and “false” findings (*red frame*)

We determined parameters for each hot spot of the computer-assisted analysis for both imaging modalities and classified these as “true” or “false” findings based on the algorithm. For classification as a “true” finding and thus “true” lymph node the finding (hot spot) had to fulfill all criteria regarding predefined SPECT and CT parameters. The program finally offered the medical experts separate lists with “true” and “false” findings for perusal and checking. The order of both lists was determined by the program based on probability of the results being “true” or “false”. The individual probabilities were calculated based both on SPECT and CT [23]. These computer-generated lists are presented in Fig. 5 as a segment from the program’s user surface as shown the lower left screen. These lists with the “true” and “false” findings are then separately evaluated and corrected by the afore-mentioned experts and, in case that certain findings are considered falsely “true” or “false” by the individual expert, they are moved to the respective other list.

A false-negative finding of the program was defined as a finding which was originally assigned to the list of “false findings” by the program, but was later assigned to the list of “true findings” by the experts. In analogy, a false-positive result was defined in the reverse manner, i. e. as a finding that was originally assigned to the list of “true findings” by the program, but was later assigned to the list of “false findings” by the experts. To this end, we matched each individual finding in our evaluation (*n* = 803), ensuring that both the number and the localization of the findings listed in the respective data analysis match. In addition, we matched our SLN diagnostic data with the information from operation reports from the Dept. of Urology and Pediatric Urology, and with the histopathological findings from the Dept. of Pathology. This matching led to surgical identification of the radio-labelled lymph nodes, proven by the fact that histological analysis detected lymphatic tissue in all samples. Each of the resected SLN corresponded to our preoperatively detected “true” findings (hot spots) according to the corrected software list. The exact workflow of the program is described by a similar workgroup around the authors of this study [23].

#### Clinical aspects: reliability and morbidity

Each enrolled patient of this study received prophylactic antibiotic therapy as a single shot application of cephalosporin prior to surgery. Incisions were made for extirpation of the lymph nodes, following the relaxed skin tension lines at the location of the marks on the skin surface. In a first step, those SLNs were resected that had preoperatively been visualized by SPECT and could intraoperatively be detected by the gamma probe. Subsequently we removed further possible radioactive LNs which were not visualized in preoperative images, but by the use of the gamma probe only. Finally an exploration of the groins followed to check for any blue-stained LNs without radioactive signal; in addition supplementary intraoperative palpation of all groins was carried out to check if suspect LNs remained. All patients were provided with drains at the end of surgery. The drains were left in place until the flow-rate was equal or smaller than 20 ml per 24 h.

All patients underwent follow-up examinations of inguinal lymph node via palpation and sonography in three-monthly intervals in accordance with the European Association of Urology (EAU) guidelines. In case of positive SLN with proven presence of malign cells, we performed classical lymphadenectomy (LAE) of the affected inguinal region, with the additional options of pelvine LAE and systematic cytotoxic therapy in a second or third step. Morbidity and disease-free survival (DFS) after SLNB were clinically evaluated during outpatient follow-up visits.

The incidence of a false-negative result of the procedure was defined as the occurrence of lymph node metastases during follow-up examinations, despite histologically inconspicuous SLN-diagnostics.

#### Statistical analysis

Sensitivity, false-negative rate, specificity and false-positive rate were evaluated statistically. We calculated the values using a cross-table and the following formulas:

$$ \mathrm{Sensitivity}=\mathrm{a}/\left(\mathrm{a} + \mathrm{c}\right) $$

$$ \mathrm{Specificity}=\mathrm{d}/\left(\mathrm{b} + \mathrm{d}\right) $$

$$ \mathrm{False}-\mathrm{positive}\ \mathrm{rate}=\mathrm{b}/\left(\mathrm{b} + \mathrm{d}\right) $$

$$ \mathrm{False}-\mathrm{negative}\ \mathrm{rate}=\mathrm{c}/\left(\mathrm{a} + \mathrm{c}\right) $$

a = Number of true-positive findings; b = Number of false-positive findings; c = Number of false-negative findings; d = Number of true-negative findings.

Prior to the procedure, all patients were duly informed about the details of the measures and gave their written informed consent for this guideline-conform procedure. In addition, the study was approved by the Ethics Committee of Kiel University (AK D 426/07).

## Results

The median age of the 25 enrolled patients at the time of primary diagnosis of the malignant disease was 60 (34–84) years. The details of the malignant disease, tumor characteristics and the results of SLN diagnostics are presented in Table 2.

**Table 2:** Tumor staging/grading of patients with results of SLNB

Tumorstaging/-grading	Patients (n)
T1	17
T2	6
T3	2
G1	2
G2	18
G3	5
Positive SLN	3
False-negative SLN	1

### Computer-assisted analysis of SPECT/CT data

The detailed results of the conventional consensual evaluation by experts, of the software alone, and of the software after correction by experts, including the required time for each, are presented for all 25 SPECT/CT datasets in Table 3.

**Table 3:** Detailed representation of results in the different assessment modes

	Column 1	2	3	4	5	6
Row 1				Conventional consensual assessment by specialists	Software assessment	Software-assessment corrected by specialists
2	Number (n)	inguinal (“true”) Lynph Nodes (LNs)	∑	83	128	83
3			R/L	47/36	65/63	42/41
4			Median	3	5	3
5			Range	0–6	0–12	0–9
6		pelvic (“true”) LNs	∑	44	87	60
7			R/L	27/17	51/36	39/21
8			Median	1	1	1
9			Range	0–8	0–13	0–11
10		all (“true”) LNs	∑	127	215	143
11			R/L	74/53	116/99	81/62
12			Median	5	6	4
13			Range	0–13	1–19	0–15
14		all (“false”) LNs	∑	-	588	660
15			Median	-	20	22
16			Range	-	3–74	3–78
17		all (“true”) and (“false”) LNs (findings)	∑	127	803	803
18			Median	5	29	29
19			Range	0–13	6–80	6–80
20	Required assessment time (min)		Average	14.6	0.91	4.4
21			Median	14.7	0.93	4.2
22			Range	12.3–17.7	0.7–1.0	2.3–7.9

The conventional consensual evaluation of 25 SPECT/CT data of lymphatic drainage scintigraphy by experts of nuclear medicine resp. radiology and nuclear medicine led to a total of 127 radio-labeled lymph nodes in 25 patients (Table 3: column 4, row 10).

The computer-based evaluation of all available data showed a total of 803 findings (hot spots), of which 215 were identified as “true” findings and 588 as “false” findings by the program (Table 3: column 5, rows 17, 10 and 14).

All radio-labelled lymph node findings detected in the expert evaluation were also identified as findings (hot spots) by the software. However, these were not always correctly allocated to the list of “true” findings, thus rendering amendments by the experts was necessary.

Thus, the findings solely generated by the software were corrected by the medical experts. As expected, the corrected evaluation of all data sets after analysis by the software still led to a total of 803 findings (hot spots), of which 143 were eventually classified as “true” findings and 660 as “false” findings (Table 3: column 6, rows 17, 10 and 14).

Table 4 shows the analysis of results of all findings regarding both lymphatic drainage regions, including the inguinal regions and the secondary drainage regions, in a crosstab. Of the 215 findings labelled “true” according to software analysis, 88 were assessed as “false-positive” by the experts and were thus attributed to the list of “false” findings. By reverse, of the findings labelled “false” (588) by software analysis, 16 were assessed as “false-negative” by the experts and thus moved to the list of “true” findings. Thus, there was a need for correction in 104 out of 803 findings. This corresponds with an overall rate of false-positive or false-negative findings of 12.95 %. In consideration of both groups of findings, both the “true” and the “false” findings in the original computer assessment, a median of 3 (0–13) corrected findings per dataset was calculated.

**Table 4:** Crosstab of the assessment results of all (inguinal and secondary) LNs, software analysis vs. correction of computer assessment by specialists (a_1_ = Number of true-positive findings; b_1_ = Number of false-positive findings; c_1_ = Number of false-negative finding; d_1_ = Number of true-negative findings)

		Correction of computer assessment by specialists		
		SLN proven	SLN disproven	Sum
Software analysis	Positive findings by software	True-positive 127 (a_1_)	False-positive 88 (b_1_)	all positive findings 215 (a_1_ + b_1_)
	Negative findings by software	False-negative 16 (c_1_)	True-negative 572 (d_1_)	all negative findings 588 (c_1_ + d_1_)
	Sum	all true SLN 143 (a_1_ + c_1_)	all false SLN 660 (b_1_ + d_1_)	all findings 803 (a_1_ + b_1_ + c_1_ + d_1_)

Considering the need for correction only in the group of originally “true” findings, a median of 2 (0–12) findings per dataset was calculated. In the group of the originally “false” findings, the median of the need for correction was 0 (0–5) findings per dataset.

When considering the inguinal regions separately, in view of the fact that it is only in the groins that radio-labelled SLN are clinically relevant in this tumor entity, then the need for correction was present in a total of 57 findings within this lymphatic drainage region. When considering both groups of findings, both the “true” and the “false” findings of the original software assessment, we calculated a median of 2 (0–8) findings per dataset for the separately considered inguinal regions that required correction.

Considering the need for correction in the separate inguinal regions only in the group of originally “true” findings, we found a median of 1 (0–7) finding per dataset with a need for correction; in the originally “false” findings we found a median of 0 (0–2) findings per dataset with a need for correction.

Based on the calculated figures and results, we were able to calculate the false-positive and false-negative rates, and thus sensitivity and specificity in a crosstab (Table 4).

The sensitivity is 88.8 % and the specificity is 86.7 %. The false-positive rate amounts to 0.133 and the false-negative rate to 0.112.

Table 5 shows the analysis of the results concerning the finding of the inguinal lymphathic drainage regions in a crosstab. However, not all cells of the crosstab can be filled as the figures are not available due to the computer analysis. Therefore we can only calculate the sensitivity value and the false-negative rate for the inguinal lymphathic drainage region.

**Table 5:** Crosstab of the assessment results of inguinal lymph nodes only, software analysis vs. correction of computer assessment by specialists (a_2_ = Number of true-positive findings; b_2_ = Number of false-positive findings; c_2_ = Number of false-negative finding; d_2_ = Number of true-negative findings)

		Correction of computer assessment by specialists		
		SLN proven	SLN disproven	Sum
Software analysis	Positive findings by software	true-positive 77 (a_2_)	false-positive 51 (b_2_)	all positive findings 128 (a_2_ + b_2_)
	Negative findings by software	False-negative 6 (c_2_)	True-negative - (d_2_)	all negative findings - (c_2_ + d_2_)
	Sum	all true SLN 83 (a_2_ + c_2_)	all false SLN - (b_2_ + d_2_)	all findings - (a_2_ + b_2_ + c_2_ + d_2_)

For the inguinal lymphatic drainage region only sensitivity is 92.8 % and in this context the false-negative rate amounts to 0.072.

The mean time span needed for the different types of assessments per patient is:

- 14.6 min for the conventional assessment by the experts
- 0.9 min for the software assessment
- 4.4 min for the correction of the software results by the experts.

### Clinical aspects: reliability and morbidity

**Fig. 6:**
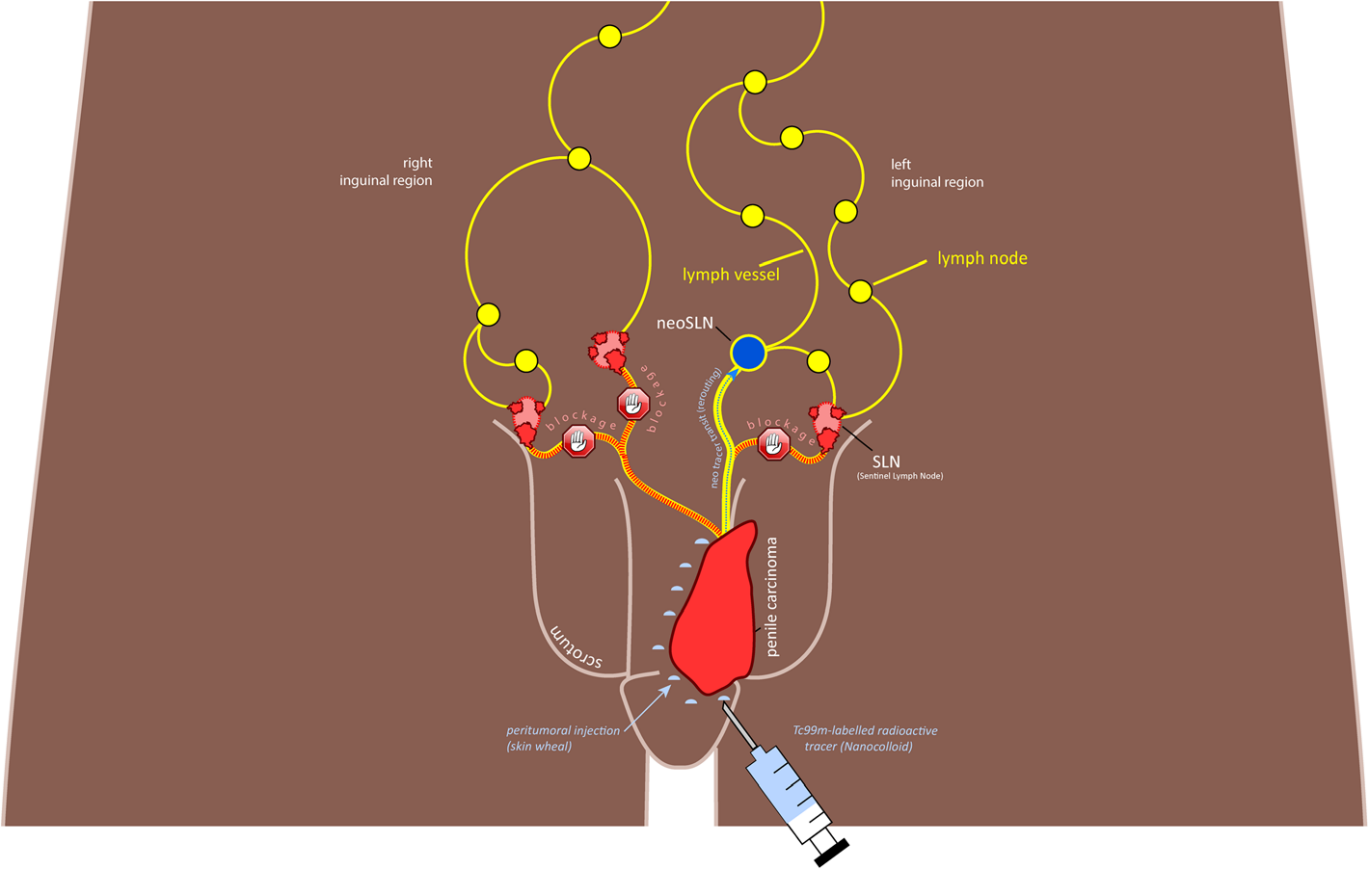
Schematic representation of inguinal lymphatic drainage after tracer application. *Right groin*: Complete blockage of tracer flow. Tumor cells (*red*) prevent drainage of the peritumorally applied tracer into the inguinal lymph nodes. *Left groin*: Rerouting of the tracer flow into the so-called neo SLN. Tumor cells in the true SLN cause rerouting of lymphatic drainage and lead to new lymphatic pathways being opened. The tracer thus accumulates in the histologically negative neo SLN

In 20 out of 25 patients the inguinal lymph node status was non-palpable and thus inconspicuous on both sides. In 5 further patients the inguinal lymph node status was palpable and thus suspect only on one side, with non-palpable lymph nodes on the other. Lymph node metastases were histologically proven in 3 out of these 5 palpable i. e. suspect groins. In the remaining 2 suspect groins the nodal status was histologically negative. One further patient showed a unilateral inguinal drainage of the tracer. The last mentioned 6 groins were not evaluated in this study as the expert society guidelines did not recommend SLNB in these cases. Thus we included 44 non-palpable, inconspicuous groins in the analysis. We removed a median number of 2 (1–5) lymph nodes per inguinal region in the groins examined by SLNB in this study. In relation to the included patients we removed a median number of 4 (1–13) lymph nodes per patient. All of the resected LNs with a blue staining also showed a radioactive signal, but not all of the radio-labelled inguinal nodes were stained. In confirmation of the preoperative palpation status, no conspicuous lymph nodes could be found during intraoperative palpation of the groins. In 3 patients lymph node metastases were diagnosed after histological SLN analysis, one per groin in each of these patients. In one patient, bilateral inguinal lymph node recurrence was diagnosed only four months after histologically negative SLNB. In this patient the tumor- and resection status was initially stated to be unclear by external experts after primary tumor excision (pT1G1Rx) ex domo. To gain clarity on the tumor- and resection stage, a re-resection of the primary tumor region was conducted in our Dept. of Urology and Pediatric Urology. After histological processing, an in sano resection (R0) with respect to the primary tumor was proven. Palpation of the groins in this patient did not show any suspect inguinal lymph nodes, so that we included this patient in the study and conducted SLNB. In the combined functional and morphological SPECT/CT imaging, we had seen a radio-labelled inguinal lymph node left, which was found to be histologically negative. In the inguinal region on the right side no tracer-labelled lymph node could be detected. Because of the fact that a bilateral inguinal lymph node recurrence occurred in this patient four months after SLNB we assume that the non-visualization of the lymph node on the right inguinal side was a consequence of a total tumor-related blockage. For the left groin, this finding can be interpreted as a so-called re-routing with visualization of a histologically negative neo-SLN (Fig. 6). This patient eventually underwent radical inguinal and pelvine LAE as well adjuvant chemotherapy. The patient has since been tumor-free during follow-up. For the other 39 groins with non-palpable inguinal lymph nodes histological analysis of SLNB led to a negative result. None of the 39 groins showed any signs of recurrence during the follow-up examinations and have remained tumor-free since SLNB. Thus these groins have shown a mean resp. Median disease-free survival of 41 resp. 43.5 months (8–79 months). From these results we calculated a false negative rate for SLNB of 4 % in relation to the number of patient (1/25 patients) as well as 4.5 % in relation to the number of examined groins (2/44 groins). In one patient a surgery-related complication in form of a prolonged inguinal lymphorrhea occurred in one groin after SLNB; the latter ceased spontaneously during Redon drainage. We calculated a morbidity rate of 2.3 % per groin (1/44 groins) resp. 4 % per patient (1/25 patients). One patient died during follow-up ca. 12 months after SLNB due to metastasized renal cell carcinoma.

Based on the data presented in Table 6 the statistical analysis per patient yields to a sensitivity of 75 % and a specificity of 100 %. The false-positive rate amounts to 0 % and the false-negative rate to 25 %. The values per groin yield to a sensitivity of 60 % and a specificity of 100 %. From this angle the false-positive rate is 0 % and the false-negative rate 40 %.

**Table 6:** Cross-table of the results of SLNB and follow-up (reference standard) and SLNB alone per patient

		Results of SLNB and follow-up (reference standard)		
		Histo. positive	Histo. negative	Sum
Results of SLNB alone	histo. positive	True-positive/a 3 (3)	False-positive/b 0 (0)	all positive findings/a + b 3 (3)
	histo. negative	False-negative/c 1 (2)	True-negative/d 21 (39)	all negative findings/c + d 22 (41)
	Sum	all true SLN/a + c 4 (5)	all false SLN/b + d 21 (39)	all findings/a + b + c + d 25 (44)

The results per groin are presented are presented in brackets

## Discussion

### Computer-assisted analysis of SPECT/CT data

As mentioned in “Background”, several studies addressing different malignant tumor entities have described the advantages of SLN scintigraphy with multimodal 3-dimensional SPECT/CT imaging for SLN diagnostics in comparison to planar scintigraphy [9–15]. But also in our study the performance of SPECT/CT SLN imaging leads to a great amount of digital data which were post-processed by a computer aided algorithm.

In our study, we calculated 88.8 % sensitivity and 86.7 % specificity based on the results of the SLN-diagnostics assessment program in 25 analyzed datasets from patients with penile cancer without palpable inguinal lymph nodes.

When considering the individual inguinal regions, which are the primary zones of lymphogenic dissemination of this tumor entity, separate analysis of the inguinal lymphatic drainage regions lead to a sensitivity value of 92.8 %.

Compared to other imaging assessment programs, our values for sensitivity and specificity are excellent. For example, Sadik et al. [25] showed the potential of computer-based assessment (EXINI bone) of nuclear medical imaging for detection of bone metastases by means of planar total bone scintigraphy. This study with a cohort of 59 patients with breast or prostate cancer, calculated a sensitivity of 90 % and a specificity of 89 % for this assessment program [25]. These results are comparable to our figures. A further study by Sadik et al. one year later showed that sensitivity of the assessing medical expert alone could be increased from 78 to 88 % by means of assistance by appropriate software. Regarding specificity, however, the aid by a program achieved no significant improvement [26]. Another study on hybrid imaging evaluated computer-assisted assessment of FDG-PET/CT data in 87 patients with suspected bronchial carcinoma. Sensitivity in this study was reported to be 86 % and specificity 100 % [27]. Further studies evaluating computer-assisted image assessment were performed for diagnostics and analysis of myocardial perfusion scintigraphy. In 2008, Tägil et al. calculated an increase of sensitivity from 81 % (without computer assistance) to 86 % with computer-assisted analysis (EXINI heart) in 97 myocard scintigraphies. Especially inexperienced physicians were able to improve their sensitivity rates with this program. In addition, the differences between the assessment of individual experts became smaller under application of the program, thus helping to reduce interobserver variability [28].

Comparison of the median values of the expert-diagnosed lymph nodes per patient with SPECT/CT imaging (5 all “true” lymph nodes/3 inguinal lymph nodes/1 secondary lymph node), with the “true” findings of Software alone per SPECT/CT dataset (6 all “true” findings/5 inguinal and 1 secondary lymph node) and the corrected “true” findings of the analysis software per SPECT/CT dataset (4 all corrected “true” findings/3 inguinal and 1 secondary finding) showed that the program in the uncorrected application suggests slightly more “true” findings as radio-labelled SLN than the experts alone or the corrected software. In practice, this could be interpreted as a higher rate of supposedly false-positive findings. The program is not fully capable on its own to reiterate the expert results, making corrections by experts necessary. Correct and accurate tracer application, which should be strictly peritumoral and intradermal, seems to have a special impact on the number of results (hot spots) offered by the program. If this measure is not carried out in an optimal way, the list of “false” findings suggested by the program will be increased, requiring more expert corrections. Categorization as “false” findings was, however, correctly accomplished in the majority of program-based data analyses.

We also evaluated the time required by this form of computer-aided data assessment; similar to the results by Baker et al. 2007, who measured a mean additional required time of 3 min 38 s for correction of computer results in a study on colorectal adenoid diagnostics via CT, the correction time required in our study was 4.4 min on average. The main factor contributing to the time consumption was, according to Baker et al., the high rate of false-positive results. Perusal and correction of these often cost more time than the confirmation of true-positive results. Correction of false-negative hot spots barely made an impact [29]. A study by Taylor et al. attempted to validate whether an increase of the false-positive rate might influence the diagnosis of the assessing expert. This was clearly disproven. Furthermore the increase of expert assessment time also became evident here, as a high rate of false-positive findings had to be checked [30]. In the current study, we were able to correct both the clearly false-positive and false-negative results of the automated assessment quickly and without any remaining doubts. Despite this, complete and careful checking of all diagnosed findings (hot spots) was necessary. This contributed, if only little, to the time expenditure, which, however, remained moderate in comparison with other studies; the time required for computer-aided image analysis per dataset with and without correction was still less than that required by conventional expert assessment, which was average 14.6 min per patient. The non-corrected, computer-assessed dataset is available after an average of 55 s, depending on computing power and speed of data input by the user. Expert validation of the suggested findings resp. lymph nodes requires 4.4 min on average according to our measurement described above. Regular application and routine could lead to further reduction of the time expenditure. It has to be noted, however, that the here applied automated assessment did not include any further morphological aspects of CT resp. classical assessment of the CT image. Supplementing functional imaging with morphological information from CT in hybrid imaging, offers the advantage of detecting unexpected, i. e. incidental clinical findings. In a study analysing the frequency of incidental findings during routine examinations, 567 out of 1426 patients (39.8 %) were diagnosed with at least one incidental finding. 55 % of these findings could be attributed to thorax CT. Six of the 567 patients experienced a clinical advantage of the incidental finding as it enabled therapy at an early stage resp. adaptation of the therapeutic regimen. Three patients, however, were unnecessarily burden through false-positive findings in this study [31]. A limiting factor of the here applied assessment program lies in the fact that it does not consider the CT-based secondary findings for SLN detection. In our patient collective, however, conventional evaluation by our medical experts did not show any case with an indication of a tumor-suspect incidental finding that might have required further investigation.

### Clinical aspects: reliability and morbidity

In our study, we were able to show excellent reliability of SLNB after application of the afore-described procedure with radio-labelled tracers for visualization of SLN, with a false negative rate of only 4.5 % per groin resp. 4 % per patient. Like us, the British workgroup around Lam et al. adopted the two-day protocol developed and first evaluated by the Horenblas workgroup. Lam and coworkers report a false negative rate of 5 % (in relation to the groins) resp. 6 % (in relation to the patients) in a cohort of 264 patients. The faulty procedures occurred chiefly during the implementation phase of the method [33].

In comparison with the results of Lam et al. [33] or Kroon et al. [34] who report rates of 28 respectively 22 % of histologically positive lymph nodes under employment of SLNB, our rate of metastatic disease is much lower (12 %). The reason might be that the patients enrolled in the study of Lam et al. had a significantly higher risk of metastatic disease, as can be seen in the lower number of T1-tumours (42 %) compared to our study (68 %). More importantly, the study of Lam et al. included a higher rate of G3-tumours (53 %). In our study the G3-tumour rate is only 20 %. A further reason is that the study by Kroon et al. included only patients with T2- and T3-tumours, while T1-tumours were not taken into account, in contrast to our study. This could explain why our values for sensitivity appear low while the false-negative rate is high. There are no data in the literature on the false negative rates of the classic inguinal staging LAE.

### Limitations of SLNB

Tumor cells can lead to obstruction resp. occlusion of the lymphatic drainage pathways, either by complete blockage or by rerouting of the radio tracer (Fig. 6) [6, 35]. Impediment of the transit resp. the drainage of the injected radioactive tracer can lead to restraints of SLNB. The risk of a relevant modification of lymphatic and thus tracer drainage depends on the metastatic load in the lymphatic pathways and in the lymph nodes. The risk of metastases in patients with palpable lymph nodes is at 50 % much higher than in those with non-palpable inguinal lymph nodes (25 %). For this reason, SLNB is not recommended by the national and international expert societies in patients with palpable inguinal lymph nodes, as mentioned before [3].

The validity of clinical groin palpation is influenced both by the physical constitution of the patient and the level of skill and experience of the examiner. In the case of the patients from our study who developed bilateral lymph node recurrence 4 months after histologically negative SLNB, the assessment of inguinal lymph node status was impeded by obesity. Obesity is capable of veiling otherwise palpable and potentially tumor-infested lymph nodes, thus wrongly leading to the conclusion that the patient is eligible for SLNB. For minimization of misjudgment we have introduced obligatory preoperative sonography of the groins in all of our patients, as a supplement to clinical palpation.

A further possible source of errors for the application of this procedure is associated with the two-stage approach of SLNB after surgical removal of primary tumor. While Graafland et al. [36] report uniform results in 40 cases of a metachronously performed SLNB, we believe that surgery-related modifications of the tracer drainage, e. g. through scar formation or edema, might be a possible cause for the false-negative SLNB in patient of this study.

### Computer-aided assessment - clinical consequences

Based on our study results, which included only one false-negative result of SLNB, we cannot answer the question whether the software in combination with fused SPECT/CT data is capable of reducing the number of false negative findings, and whether the patients can expect a diagnostic and prognostic advantage regarding morbidity and especially mortality. In the case of a false-negative SLNB, which showed a bilateral lymph node recurrence four months after histologically inconspicuous SLNB, application of the software had failed to visualize a hotspot due to complete tracer blockage in the right inguinal region, and on the left side a so-called re-routing with visualization of a neo-SLN occurred (Fig. 6), which was also visualized in the tracer image of computer-assessment, but turned out to be histologically negative. Additional imaging with FDG-PET/CT is capable of improving preoperative diagnostics with a particular view to cases with palpable lymph nodes or a doubtful lymph node status.

Despite consensus reading by experienced physicians, the expert analysis alone (n = 127) missed a few hot spots when compared against the total number of lymphatic drainage regions detected and offered in lists of true and false findings by the program (Fig. 5).

Consensus analysis by experienced physicians alone yielded 127 hot nodes in all lymphatic drainage regions. After expert correction of the program results, the number of the detected hot spots in all lymphatic regions increased to 143 (Table 3, line 10). Thus 16 hot spots were missed when comparing both types of analyses; the missed hot spots were exclusively located in the pelvic lymphatic regions. In view of the high sensitivity of the software, we assume that the experts were able to make their decision regarding the higher number of findings suggested by the program with greater confidence. But the number of hot nodes detected in both analyses was the same (*n* = 83, Table 3, line 2), when focusing on the interesting inguinal regions only. Therefore the surgical procedures were not influenced significantly by the results of the computer analysis, as the LN surgery, as part of the SLNB procedure, was strictly limited to the groins. Despite the fact that the images and their interpretation were available prior to surgery for surgical planning, intraoperative detection of radioactive lymph nodes using a gamma probe lead to nearly the same number of detected radioactive lymph nodes in our 25 patients (83 vs. 88/94.3 %). The crucial aspect is that all preoperatively detected hot nodes in both types of preoperative iamge evaluation were found intraoperatively by using the probe. Intraoperative use of the sensitive gamma probe also lead to the detection of 5 additional radioactive lymph nodes in our 44 investigated groins. We assume that the direct identification of the radioactive inguinal lymph nodes using the handheld probe at a negligible distance and the absence of tissue attenuation during surgery, which is present in the preoperative scintigraphical imaging, lead to a higher sensitivity of the intraoperative probe, resulting in a higher number of detected lymph nodes (88). Moreover, the time span between pre- and intraoperative SLN detection may have led to a more extensive tracer flow in the lymphatic pathways and may also be responsible for the higher number of intraoperatively detected inguinal lymph nodes in this study.

Application of computer-aided assessment in larger patient collectives, and possibly also in other tumor entities, will have to show whether the software can help to further reduce the low recurrence rates, based on the fact that computer-aided assessment tends to indicate a higher number of potential metastatic lymph nodes for radio-guided biopsy.

Currently, the concept behind the software is to distinguish between “true” and “false” findings exclusively on the basis of pre-defined parameters in the program. Correction of individual findings is both necessary and possible. Potential improvements are currently not processed by the program and thus cannot be applied in the succeeding datasets, in other words, the program cannot learn from the expert corrections. A self-improving program would be capable of continuously minimizing the number both of false-positive and false-negative results based on supervised “Machine-Learning” approaches. In this context, so-called “artificial neural networks” (ANN) play a role as they may be able to remedy current limitations.

There are data on the impact of ANN on existing assessment software, regarding the reduction of false-positive findings. Thus a program for detection of pulmonary coin lesions was able to reduce the rate of false-positive findings by more than 68 % after an ANN algorithm had been established, without impairing the sensitivity of the procedure [32].

Implementation of a similar algorithm would be advantageous for the assessment program examined here, and will be in focus during further development of the system.

At the current stage, the program is not yet capable of performing fully automatic and correct assessment of the SPECT/CT datasets (especially with regard to further assessment of the CT-data as described in “Methods”). False-positive and false-negative findings continue to require expert correction, ideally by experts with combined knowledge in nuclear medicine and radiology. While the assessment software evaluated here cannot replace expert assessment after preoperative SLN diagnostics in penile cancer, it can supplement and support the expertise even of highly specialized physicians. Moreover, the program is an apt tool for supporting and educating doctors in training and can help them to double-check their own assessment of findings.

## Conclusions

SLNB with SLN-labelling by means of Tc-99m nanocolloids in penile cancer is a valuable diagnostic method with low morbidity. It offers a high degree of reliability, especially when it is performed during resection of the primary tumor. The here evaluated computer-assisted assessment of SPECT/CT data for SLN diagnostics shows high sensitivity and specificity in this tumor entity. While the program cannot replace expert assessment, it is able to provide substantial support and relief for the medical experts. Especially less experienced doctors and specialists in training are able to profit from this program during their learning curve. However, the methodical and logistic demands placed to an interdisciplinary team remain high despite (semi-) automated data assessment. This method should only be applied in specialized centers and by interdisciplinary teams in this rare tumor entity.

## Abbreviations

ANN: Artificial neural networks
CT: Computer tomography
DFS: Disease-free survival
EAU: European Association of Urology
FDG-PET/CT: Fluor-deoxyglucose positron-emission-computed-tomography/computertomography
HU: Hounsfield units
IT: Information technology
LAE: Lymphadenectomy
LEHR: Low energy high resolution
MIP: Maximum-intensity-projection
MRI: Magnetic resonance imaging
SLNB: Sentinel lymph node-biopsy
SLN: Sentinel lymph node
SPECT/CT: Single photon emission computed tomography/computed tomography
SPM: Statistical parametric mapping
μm: Micrometer
